# Hepatospecific ablation of p38α MAPK governs liver regeneration through modulation of inflammatory response to CCl_4_-induced acute injury

**DOI:** 10.1038/s41598-019-51175-z

**Published:** 2019-10-10

**Authors:** Manon Fortier, Mathilde Cadoux, Nadia Boussetta, Sandrine Pham, Romain Donné, Jean-Pierre Couty, Chantal Desdouets, Séverine Celton-Morizur

**Affiliations:** 1 Centre de Recherche des Cordeliers, INSERM, Sorbonne Université, USPC, Université Paris Descartes, Université Paris Diderot, Team (Proliferation, Stress and Liver Physiopathology), F-75006 Paris, France; 2 Institut Cochin, INSERM U1016, CNRS UMR8104, Paris Descartes University, Paris, France

**Keywords:** Cell death and immune response, Hepatotoxicity

## Abstract

Mammalian p38α MAPK (Mitogen-Activated Protein Kinase) transduces a variety of extracellular signals that regulate cellular processes, such as inflammation, differentiation, proliferation or apoptosis. In the liver, depending of the physiopathological context, p38α acts as a negative regulator of hepatocyte proliferation as well as a promotor of inflammatory processes. However, its function during an acute injury, in adult liver, remains uncharacterized. In this study, using mice that are deficient in p38α specifically in mature hepatocytes, we unexpectedly found that lack of p38α protected against acute injury induced by CCl_4_ compound. We demonstrated that the hepatoprotective effect alleviated ROS accumulation and shaped the inflammatory response to promote efficient tissue repair. Mechanistically, we provided strong evidence that Ccl2/Ccl5 chemokines were crucial for a proper hepatoprotective response observed secondary to p38α ablation. Indeed, antibody blockade of Ccl2/Ccl5 was sufficient to abrogate hepatoprotection through a concomitant decrease of both inflammatory cells recruitment and antioxidative response that result ultimately in higher liver damages. Our findings suggest that targeting p38α expression and consequently orientating immune response may represent an attractive approach to favor tissue recovery after acute liver injury.

## Introduction

Acute liver injuries (ALI) can be caused by drug, virus, alcohol, toxic chemical, and several other factors and is a common pathway to many liver diseases^1–5^. The pathogenesis of ALI involves inflammation, oxidative stress coupled to the production of reactive oxygen species (ROS) and hepatocyte cell death (apoptosis and necrosis)^6–9^. ALI are characterized by a rapid resolution and a complete restitution of normal organ architecture and function after the elimination of the cause. However, in some cases, ALI may progress to chronic liver injury, hepatic fibrosis, or even hepatocellular carcinoma^10,11^. Therefore, searching for new therapeutic strategies improving recovery process is critical for a better handling of liver diseases.

p38 Mitogen-activated protein kinases (MAPKs) are essential for the cellular response against injury by integrating a plethora of pathways including growth, inflammation, metabolism and apoptosis^12–14^. Among all p38 isoforms, p38α (MAPK14) is the best characterized and expressed in most cell types^15^. As mice lacking p38α isoform die in utero due to angiogenic defects in the placenta and peripheral vessels^16–18^, mice models harboring tissue-specific deletion of p38α have been developed. During liver regeneration following partial hepatectomy, mice with specific ablation of p38α in hepatocytes early in life exhibited enhanced hepatocyte proliferation revealing that p38α acts as an inhibitor of hepatocyte proliferation by antagonizing the activity of the JNK–c-Jun pathway^19–21^. By contrast, liver-specific ablation of p38α during chronic biliary cirrhosis reduced hepatocyte cell growth, caused mitotic blockade and cytokinesis failure impairing dramatically mice lifespan^22^. Studies in thioacetamide (TAA) and DiEthyl-Nitrosamine (DEN)-induced HCC mice models revealed that p38α acts as a tumor suppressor by curtailing ROS accumulation protecting against cell death, subsequent compensatory hepatocyte proliferation and liver tumor development^23–25^. Collectively, these studies highlight that p38α displays several functions that critically depend on the physiopathological context. However, the impact of p38α deletion during acute liver injury in completely mature adult hepatocytes is still an open question.

In that context, to determine the role of p38α in the adult liver, we developed a mice model allowing the deletion of p38α in mature hepatocytes. Using acute liver injury model, our findings reveal quite unexpectedly that p38α deletion is translated into a potent hepatoprotective response against liver injury. Interestingly, we demonstrated that p38α deficiency instructs the inflammatory response to promote efficient tissue repair.

## Results

### p38α deletion protects mice against acute hepatocellular damage

**Figure 1:**
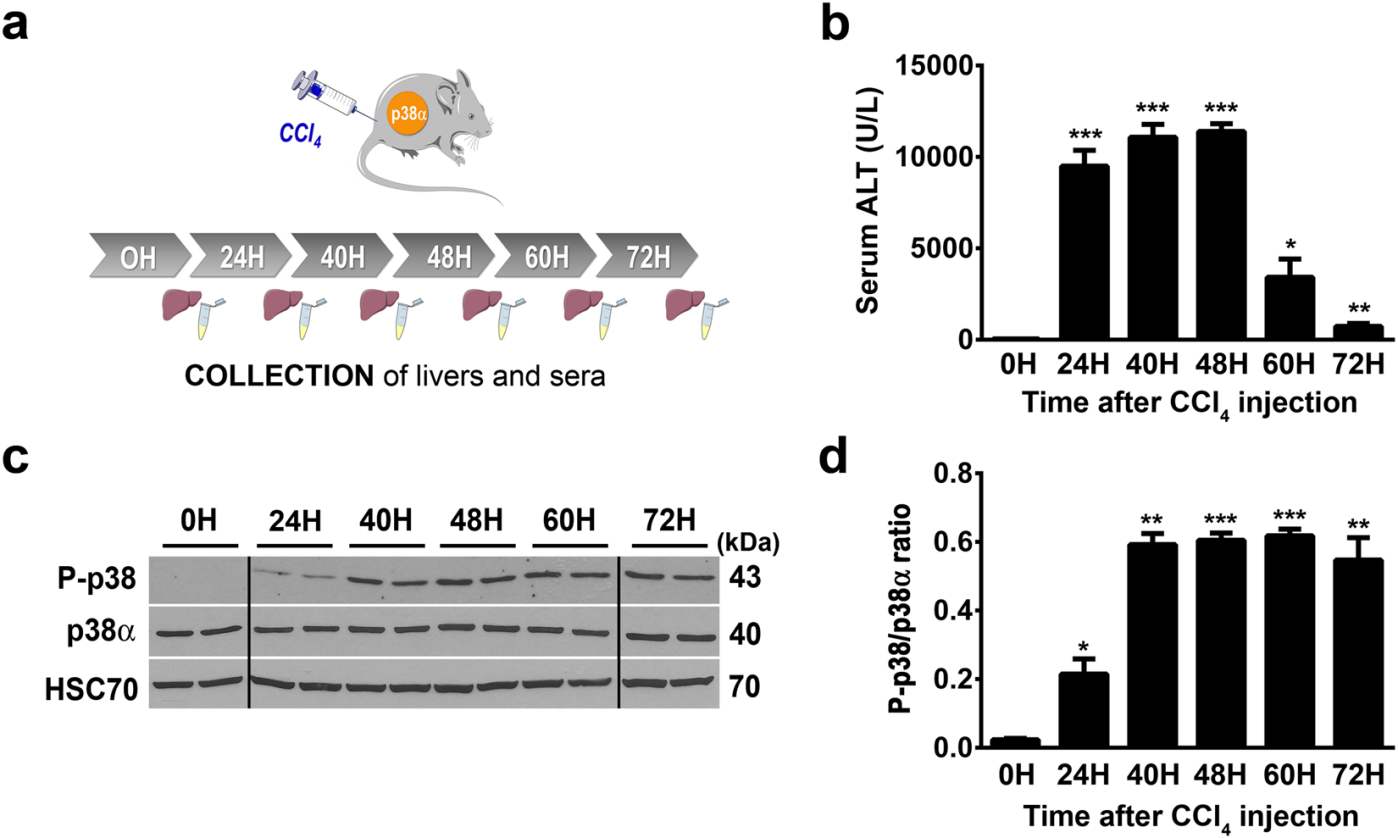
p38α is activated during acute CCl_4_ exposure. (**a**) Schematic representation of experimental procedure for CCl_4_ injection in control mice (CTR). (**b**) Average alanine aminotransferase (ALT) levels in CTR sera samples before (0H) and after (24H to 72H) CCl_4_ exposure. Data represent the mean ± SEM (*n* ≥ 6 per group); *p < 0.05, **p < 0.01, ***p < 0.001 (two-tailed t-test), as compared to 0H. (**c**) Phospho-p38 and p38α expression in liver of CTR mice before (0H) and after (24H to 72H) CCl_4_ exposure. HSC70 served as a loading control. Lanes showed samples from independent biological replicates and were noncontiguous (black line). The displayed figure was cropped and the original images are part of the Supplementary Data. (**d**) Densitometry analysis of P-p38 vs p38α protein levels before (0H) and after (24H to 72H) CCl_4_ injection. Data represent the mean ± SEM (*n* ≥ 3 per group). *p < 0.05, **p < 0.01, ***p < 0.001 (two-tailed t-test) as compared to 0H.

**Figure 2:**
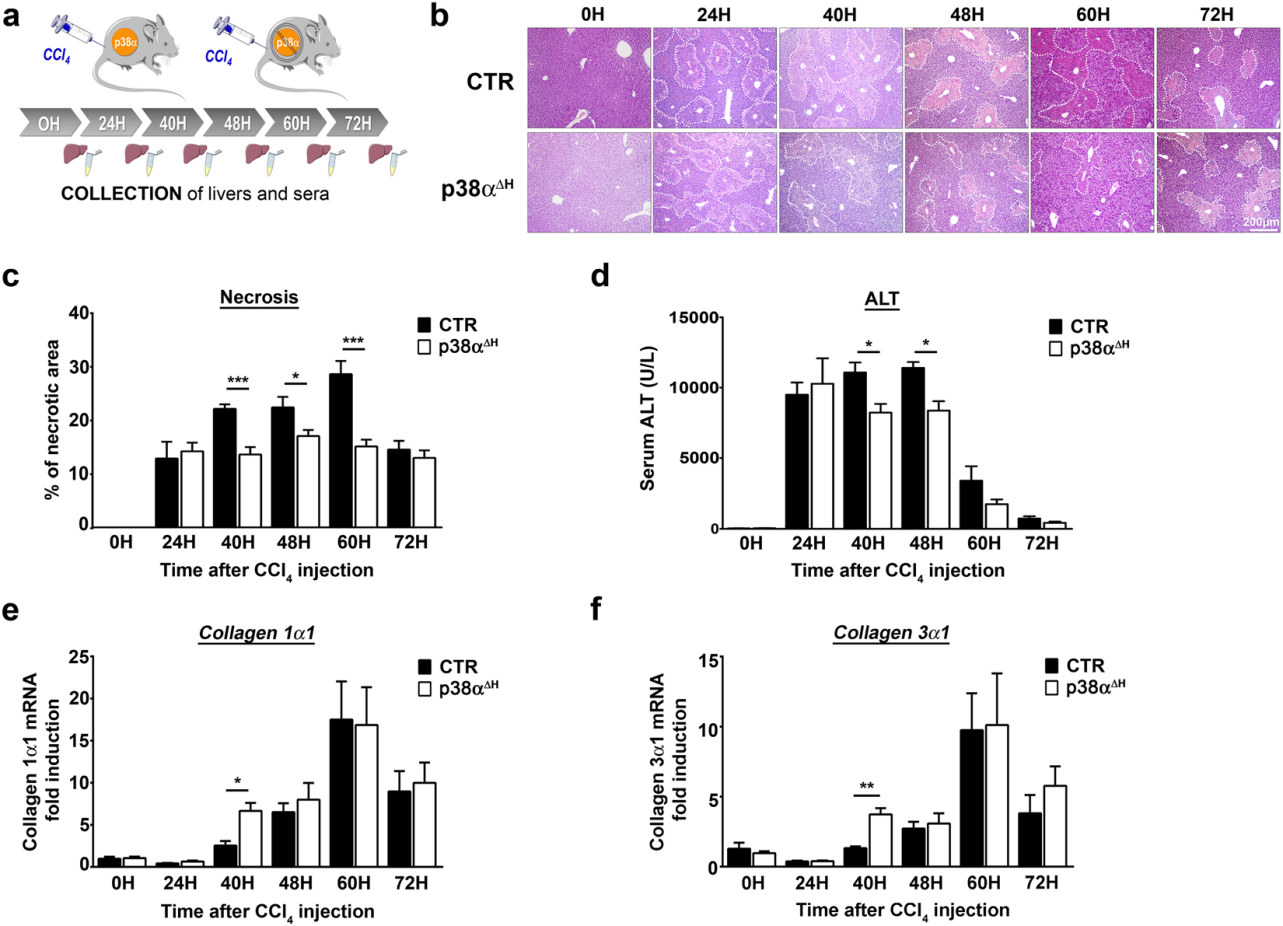
p38α ablation in mature hepatocyte is hepatoprotective against CCl_4_-induced liver injury. (**a**) Schematic representation of experimental procedure for CCl_4_ injection in control mice (CTR) and p38α^ΔH^ mice. (**b**) Representative haematoxylin and eosin (H&E) staining of liver tissue sections from CTR and p38α^ΔH^ mice at different time points after CCl_4_ injection. (**c**) Quantification of necrotic area from H&E stained CTR and p38α^ΔH^ liver sections at indicated time points after CCl_4_ injection. Data represent the mean ± SEM (*n* ≥ 7 per group); *p < 0.05, ***p < 0.001 (two-tailed t-test). Average alanine aminotransferase (ALT) levels in CTR and p38α^ΔH^ sera samples at indicated time points after CCl_4_ injection. Data represent the mean ± SEM (*n* ≥ 6 per group); *p < 0.05 (two-tailed t-test). (**e,f**) Relative mRNA level of *Collagen 1α1* (**e**) and *Collagen 3α1* (**f**) measured by quantitative PCR in CTR and p38α^ΔH^ liver samples at indicated time points after CCl_4_ injection. Gene expression levels were normalized to the abundance of *18s* mRNA for each sample. Data represent the mean ± SEM (*n* ≥ 6 per group); *p < 0.05, **p < 0.01 (two-tailed t-test).

Acute administration of carbon tetrachloride (CCl_4_), is widely used in experimental animal models of liver failure that mimics human hepatic response against toxic compounds^26,27^. CCl_4_ is a strong hepatotoxin that induces overproduction of ROS, lipid peroxidation of membranes, causes hepatocyte death and inflammation, resulting to severe hepatotoxicity^28,29^. Protection against apoptosis, inflammation and oxidative stress associated with a pro-regenerative response of the hepatocytes are crucial to ensure efficient tissue repair after detrimental CCl_4_ exposure. First, to evaluate the activity of p38α during acute liver injury, control mice were injected by a single dose of CCl_4_ and liver and sera were collected during time course kinetic (Fig. 1a). A single-dose of CCl_4_ induced significant liver injuries reflected by hepatocyte cytolysis that we monitored by the evaluation of ALT (Alanine Transaminase) plasma level (Fig. 1b). Indeed, ALT level picked from 24 and 48 hours (injury phase) post-injection of CCl_4_ and gradually decreased at 60 and 72 hours (recovery phase) (Fig. 1b). In that context, we investigated the profile of p38α phosphorylation/activation in the injured liver. To that end, p38α and Thr180/Tyr182 phospho-p38 protein levels were measured by western blot analysis in a time course experiment (Fig. 1c,d). We first observed that the expression of p38α was stable all along the kinetic (Fig. 1c). Second, whereas in the resting liver (0H) we detected a weaker signal of P-p38, the phosphorylation of p38 increased gradually after CCl_4_ exposure, concomitant with the increasing tissue injury observed in the liver (Figs 1b and 2b,c) and reached a plateau at 40 hours until the end of the kinetic (Fig. 1c,d). These findings indicated that acute liver injury mediated by CCl_4_ exposure induced specific activation of p38α.

To better characterize the role of p38α during CCl_4_ injury, hepatocyte-specific deletion of p38α (p38α^ΔH^) was achieved by crossing mice carrying conditional loxP-flanked p38α alleles (p38α^fl/fl ^^30^;) with transgenic mice expressing the Cre recombinase under the control of the hepatospecific transthyretin promoter (TTR-Cre Tam^31^;). Tamoxifen diet induces very efficient ablation of p38α expression in the liver of p38α^ΔH^ mice even though some remaining expression of p38α was visible due to the presence of nonparenchymal cells that are not targeted by the TTR-Cre transgene (Supplementary Fig. 1a). Interestingly, following p38α hepatospecific deletion (p38α^ΔH^) and under steady state conditions, we did not observe any signs of alterations within liver parenchyma. From these results, we concluded that p38α expression in adult hepatocyte is not absolutely required to maintain liver homeostasis during steady-state conditions.

**Figure 3:**
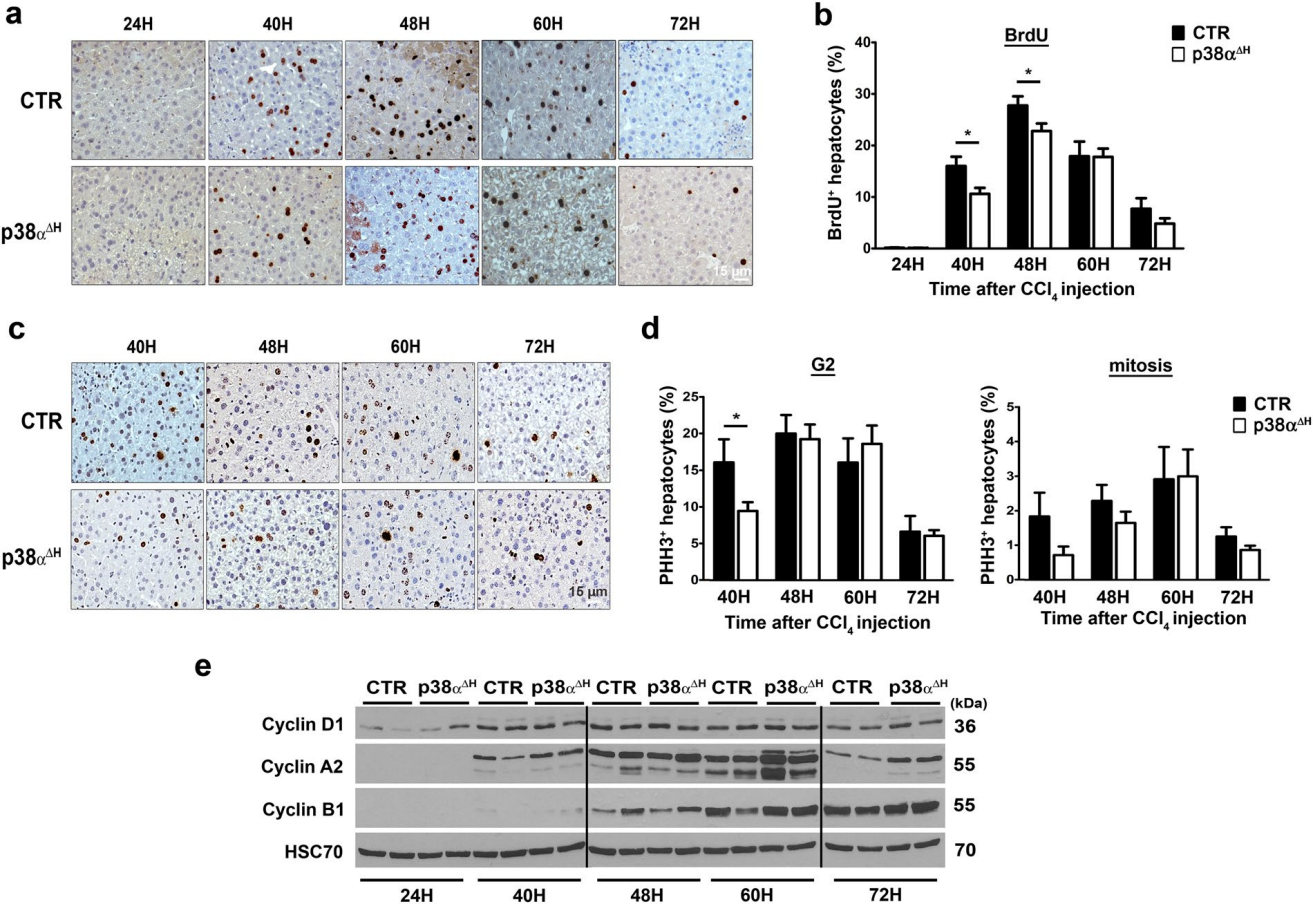
p38α deficiency does not favor hepatocyte proliferation during acute injury. (**a**) Representative BrdU immunochemistry of control (CTR) and p38α^ΔH^ liver tissue at indicated time points after CCl_4_ injection. (**b**) Quantitative analysis of BrdU labeled CTR and p38α^ΔH^ liver sections (percentage of BrdU^+^ hepatocytes). Data represent the mean ± SEM (*n* ≥ 6 per group); *p < 0.05 (two-tailed t-test). (**c**) Representative Phospho-Histone H3 (PHH3) immunochemistry of CTR and p38α^ΔH^ liver tissue at indicated time points after CCl_4_ injection. (**d**) Quantitative analysis of PHH3^+^ hepatocytes in G2 phase (left panel) and mitosis (right panel) in CTR and p38α^ΔH^ livers (histologic distinction of PHH3^+^ hepatocytes). Data represent the mean ± SEM (*n* ≥ 6 per group). *p < 0.05 (two-tailed t-test). (**e**) Immunoblot of proteins regulating cell cycle progression (Cyclin D1, A2 and B1) in CTR and p38α^ΔH^ liver samples at indicated time points after CCl_4_ injection. Two representative samples are shown for each analyzed point. HSC70 served as a loading control. Lanes were noncontiguous (black line). The displayed figure was cropped and the original images are part of the Supplementary Data.

p38α^ΔH^ mice and their respective controls were challenged by a single CCl_4_ injection and we monitored in time the hepatocyte cytolysis and liver damage (Fig. 2a). H&E staining of liver sections indicate that significant necrosis was already present from 24 hours in the liver of p38α^ΔH^ and control mice (Fig. 2b,c, Supplementary Fig. 1b). Interestingly, necrotic areas increased gradually and peaked at 60 hours post-CCl_4_, to diminish at 72 hours in the liver of control mice (Fig. 2b,c). However, although necrotic areas were still evident between 40 and 48 hours in the liver of p38α^ΔH^ mice, the intensity of necrosis was markedly reduced as compared to the controls (Fig. 2b,c). Accordingly, ALT levels in p38α^ΔH^ mice remained strictly lower compared to control mice at these time points (Fig. 2d). Furthermore, cleaved caspase-3 staining was used to examine apoptosis of hepatocytes in both group of mice at 24, 40 and 48 hours post-CCl_4_ treatment (Supplementary Fig. 1c). Our observations revealed that apoptotic response consecutive to CCl_4_ challenge was not impaired in p38α^ΔH^ liver and could not account for the decrease of both necrotic areas and ALT levels observed in p38α-related deficiency context (Supplementary Fig. 1c,d). To rule out the possibility that differential CCl_4_ bioactivation could be responsible for the variation in the liver injury between control and p38α^ΔH^ mice, we measured mRNA level of cyp2e1, a major CCl_4_-metabolizing enzyme. First, we did not find difference in the mRNA level of cyp2e1 under steady state conditions between both groups of mice (Supplementary Fig. 1e). Moreover, consistent with previous reports^32,33^, the CCl_4_ treatment resulted in a decrease of cyp2e1 mRNA level between 12 and 24 hours, indicating the same metabolization process of CCl_4_ compound in both groups of mice (Supplementary Fig. 1e). Interestingly, we monitored collagens 1α1 (Fig. 2e) and 3α1 (Fig. 2f) mRNA levels and we found an up-regulation at 40 hours in the liver of p38α^ΔH^ mice as compared to control one (Fig. 2e,f), reflecting an earlier tissue repair response under p38α deficiency. These findings suggest that p38α ablation in adult hepatocytes both buffers liver injury and favors a better response in tissue recovery.

### Proliferative response induced by acute CCl_4_ was not affected by p38α ablation

Since p38α MAPK has been largely reported as a negative regulator of cellular proliferation controlling the induction of both G1/S and G2/M cell cycle checkpoints^34–37^, we checked the consequences of p38α deficiency on hepatocyte proliferative response after CCl_4_ exposure. We monitored bromodeoxyuridine (BrdU) incorporation (Fig. 3a) in both control and p38α^ΔH^ livers during time-course kinetic. BrdU-positive hepatocytes were detected as soon as 40 hours post-CCl_4_ and the percentage of BrdU-positive hepatocytes peaked at 48 hours, to gradually decrease afterward, in both mice groups (Fig. 3b). Contrary to what we expected, we did not observe a global enrichment of BrdU-positive hepatocytes in p38α^ΔH^ livers. In fact, BrdU immune-reactive cells were modestly decreased in p38α^ΔH^ livers compared to control livers at 40 and 48 hours post-CCl_4_ exposure (Fig. 3b). To reinforce these interesting results, we analyzed G2 phase and mitosis progression using PHH3 labeling (Fig. 3c,d). In lines with the assessment of BrdU analysis in p38α^ΔH^ livers, the percentage of PHH3-positive hepatocytes was slightly reduced at 40 hours after CCl_4_ exposure compared to control livers (Fig. 3d). Moreover, molecular analysis of key drivers of cell cycle progression (cyclin D1 (G1 phase), A2 (S phase) and B1 (G2/M)), did not reveal significant differences between the two groups of mice (Fig. 3e). Altogether, these data revealed that p38α deficiency does not impact on hepatocyte cell cycle during acute injury. Importantly, our findings revealed that the hepatoprotective response driven by p38α deletion is largely independent of its known role of cell cycle checkpoint.

### Enhancement of antioxidative response protect against CCl_4_-mediated injury in the absence of p38α

Since CCl_4_ causes severe liver cell damages through a strong elevation of oxidative stress response^38,39^ and that p38α is a mediator of the cellular redox balance in hepatocytes^24,25,40^, we tested whether the hepatoprotective effect observed after p38α ablation could be attributable to an enhancement of the antioxidative response.

**Figure 4:**
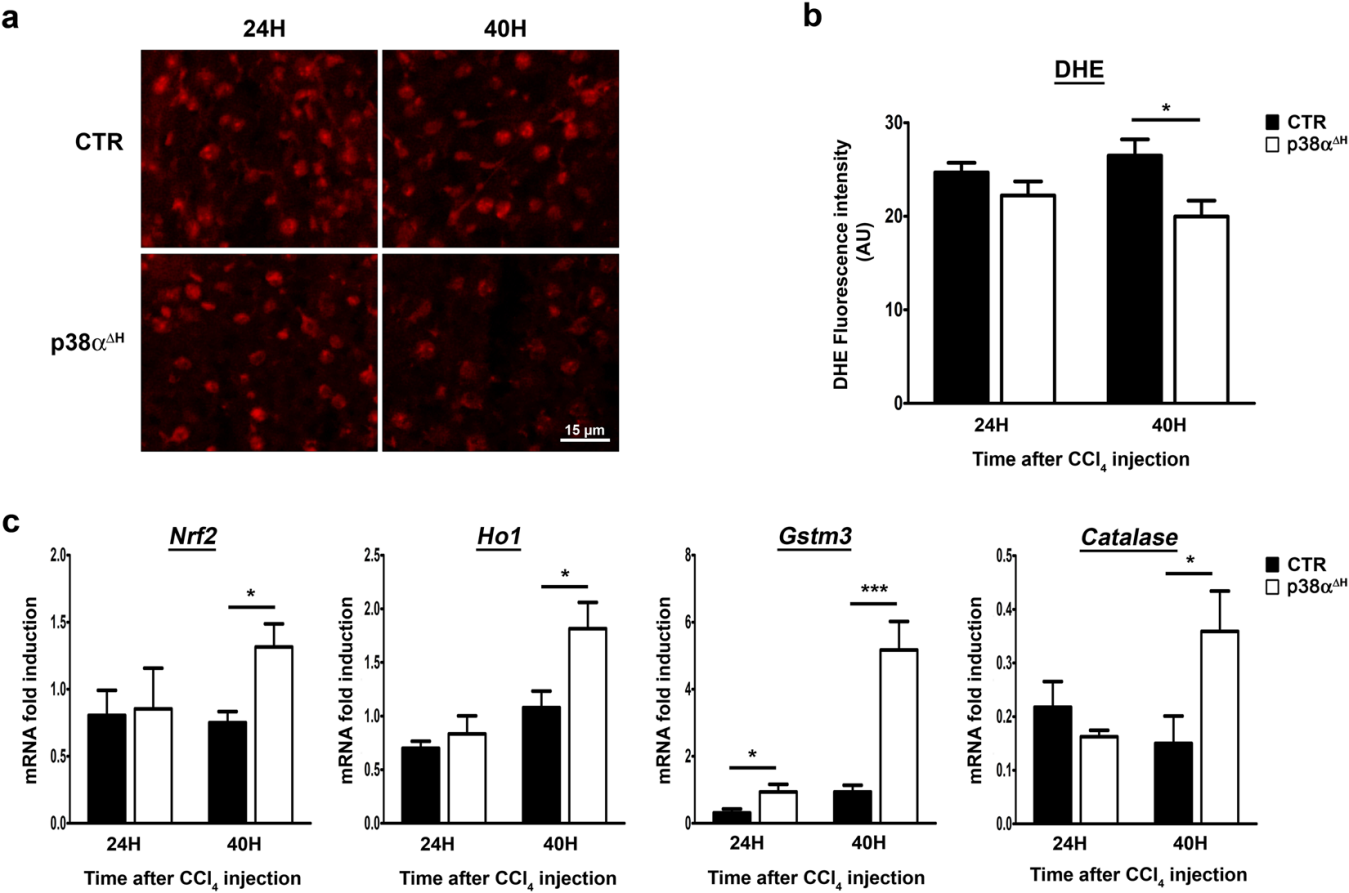
Enhancement of the anti-oxidant response in the liver of p38α^ΔH^ mice after acute CCl_4_ exposure. (**a**) Representative images of Dihydroethidium (DHE) staining of control (CTR) and p38α^ΔH^ liver sections at 24 and 40 hours post-CCl_4_. (**b**) Quantification of DHE fluorescence intensity (arbitrary unit) obtained from staining of CTR and p38α^ΔH^ liver sections at 24 and 40 hours post-CCl_4_. Data represent the mean ± SEM (*n* ≥ 5 per group). *p < 0.05 (two-tailed t-test). (**c**) Relative mRNA level of antioxidant genes (*Nrf2*, *Ho1*, *Gstm3* and *Catalase*) measured by quantitative PCR in CTR and p38α^ΔH^ liver samples at 24 and 40 hours post-CCl_4_. Gene expression levels were normalized to the abundance of *18s* mRNA for each sample. Data represent the mean ± SEM (*n* ≥ 6 per group). *p < 0.05, ***p < 0.001 (two-tailed t-test).

The general level of hepatic ROS was assessed by the fluorescent dye dihydroethidine (DHE) on fresh frozen liver sections from both control and p38α^ΔH^ mice (Fig. 4a,b). Whereas no significant differences in ROS levels were observed at 24 hours post-CCl_4_ in the liver of both p38α^ΔH^ and control mice, we did notice, at 40 hours, that ROS accumulation was largely decreased in p38α^ΔH^ livers (Fig. 4a,b). This observation indicated that p38α deficiency dampened oxidative stress. To gain insights into the signaling pathway, we investigated the Nrf2-mediated signaling as an essential component for the inhibition of oxidative stress in mice during acute liver injury^41–43^. Interestingly, we found that Nrf2 transcripts (Fig. 4c) and its downstream effectors Ho-1, Catalase and Gstm3 (Fig. 4c) were significantly enhanced in p38α^ΔH^ compared to control livers at 40 hours post-CCl_4_ injury. These findings suggested that p38α deficiency is translated into a protective effect against CCl_4_-induced ROS formation at least through Nrf2 pathway.

### p38α deletion impacts on the inflammatory response during acute liver injury

**Figure 5:**
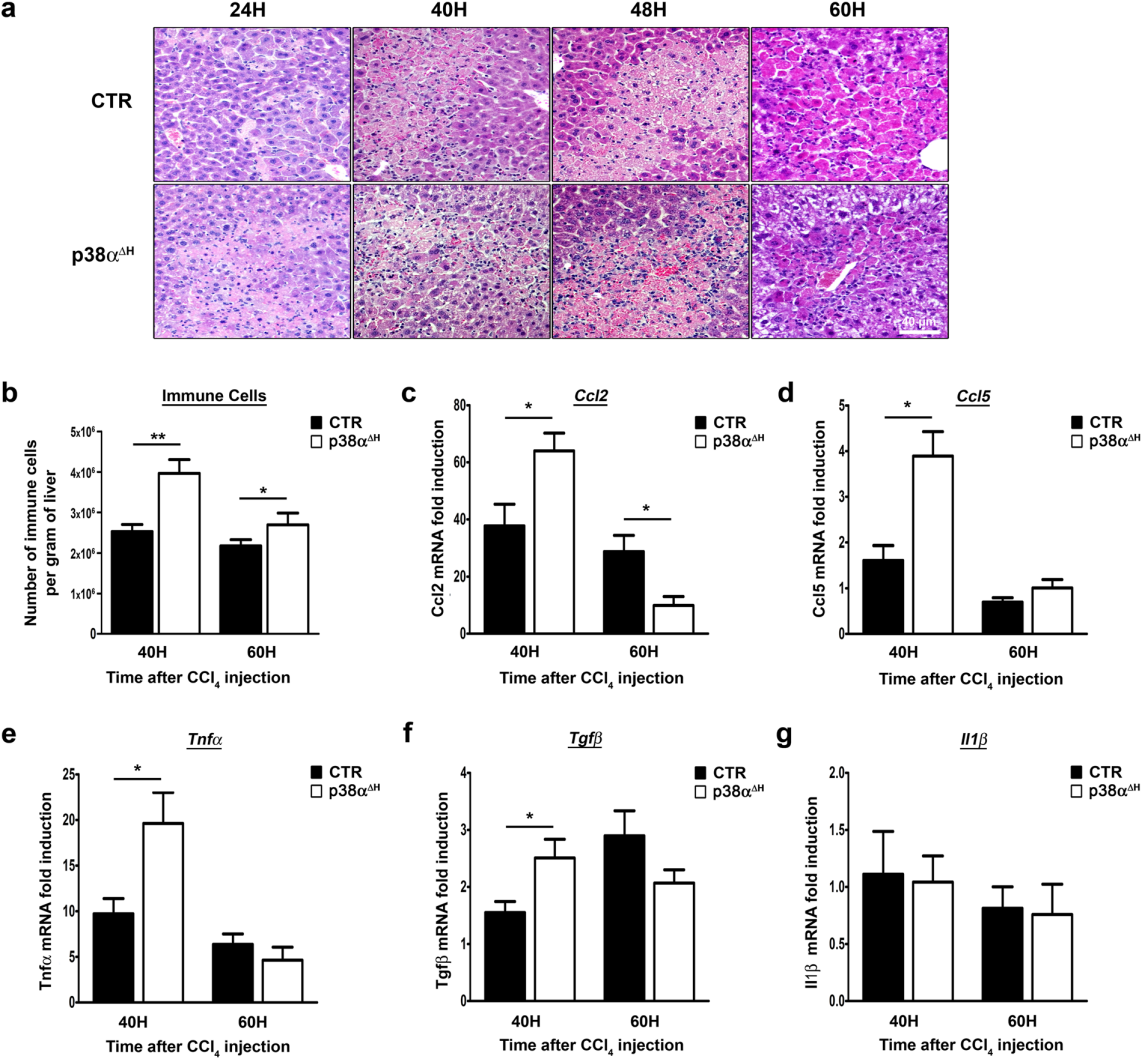
p38α deficiency instructs the inflammatory response to promote efficient tissue repair.(**a**) Representative photomicrographs of immune infiltration with H&E staining in control (CTR) and p38α^ΔH^ liver sections at different time points after CCl_4_ injection. (**b**) Number of hepatic immune cells per gram of liver in CTR and p38α^ΔH^ mice at 40 and 60 hours post-CCl_4._ Data represent the mean ± SEM (*n* ≥ 5 per group). *p < 0.05, **p < 0.01 (two-tailed t-test). (**c–g**) Relative mRNA level of *Ccl2, Ccl5, Tnfα, Tgfβ and Il1β* measured by quantitative PCR in CTR and p38α^ΔH^ liver samples at indicated time points after CCl_4_ injection. Gene expression levels were normalized to the abundance of *18s* mRNA for each sample. Data represent the mean ± SEM (*n* ≥ 5 per group). *p < 0.05 (two-tailed t-test).

Interestingly, at the level of H&E staining, we observed along the kinetic read-out, a substantial increase of inflammatory cells within necrotic areas in p38α^ΔH^ compared to control livers (Fig. 5a). We then extracted immune cells from the livers and confirmed their increase in p38α^ΔH^ mice compared to control mice at 40 hours after CCl_4_ injury (Fig. 5b). Interestingly, at 60 hours, the number of immune cells decreased in p38α^ΔH^ liver but still remained higher than in control liver (Fig. 5b). To go further, we monitored chemotactic signals, which play an essential role during acute liver injury by managing the migration of immune cells^44^. We found a significant up-regulation of both Ccl2 (Fig. 5c) and Ccl5 (Fig. 5d) at 40 hours post-CCl_4_ challenge, suggesting that these chemokines favor the drastic immune cell recruitment in p38α^ΔH^ livers.

Next, we evaluated common genes involved in inflammation on whole liver tissue from both groups of mice. Importantly, at 40 hours post-injury, we found a concomitant up-regulation of *Tnfα* (Fig. 5e) and *Tgfβ* (Fig. 5f) expression without modifications in *Il1β* mRNA level (Fig. 5g) suggesting a particular inflammatory flavor sustaining tissue repair. Altogether, our data suggested that the increase in immune cells could be involved into the hepatoprotective response driven by p38α ablation.

**Figure 6:**
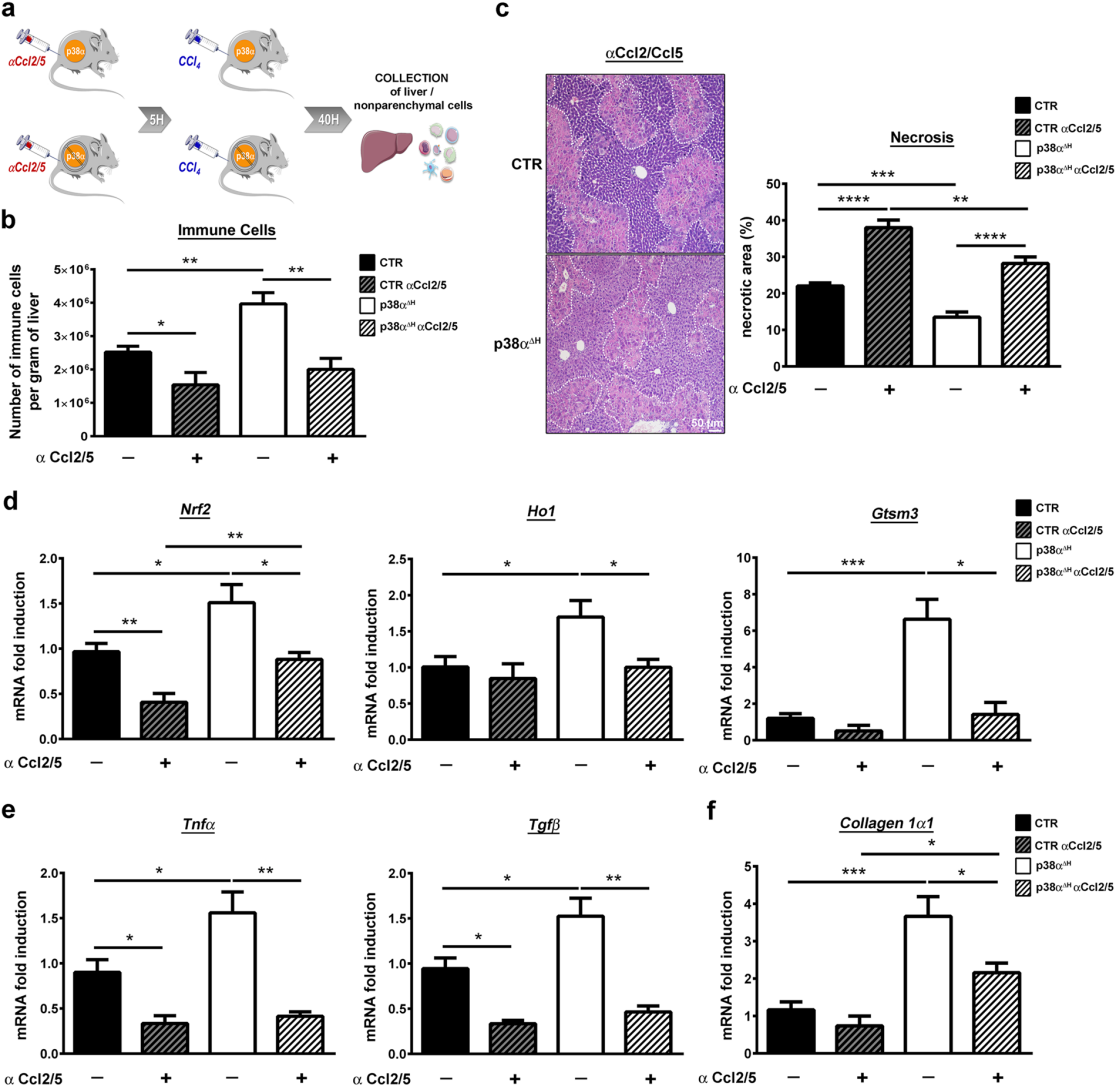
Blockade of Ccl2/Ccl5 chemotactic signals impairs hepatoprotective effect coupled to p38α deficiency during acute liver injury. (**a**) Schematic representation of experimental procedure for Ccl2 and Ccl5 blockade. Control (CTR) and p38α^ΔH^ mice were sacrificed at 40 hours after CCl_4_ injection. (**b**) Number of immune cells per gram of liver in CTR and p38α^ΔH^ mice treated or not by Ccl2/Ccl5 antibodies, 40 hours after CCl_4_ exposure. Data represent the mean ± SEM (*n* ≥ 5 per group). *p < 0.05, **p < 0.01 (two-tailed t-test). (**c**) Necrotic area analysis by liver section H&E staining of CTR and p38α^ΔH^ mice treated or not by Ccl2/Ccl5 antibodies and its quantification at 40 hours post-CCl_4_. Data represent the mean ± SEM (*n* ≥ 5 per group). **p < 0.01, ***p < 0.001, ****p < 0.0001 (two-tailed t-test). (**d**) Relative mRNA level of antioxidant genes (*Nrf2*, *Ho1* and *Gstm3*) measured by quantitative PCR in CTR and p38α^ΔH^ livers issued from mice treated or not by Ccl2/Ccl5 antibodies and its quantification at 40 hours post-CCl_4_. Gene expression levels were normalized to the abundance of *18 s* mRNA for each sample. Data represent the mean ± SEM (*n* ≥ 3 per group). *p < 0.05, **p < 0.01, ***p < 0.001 (two-tailed t-test). (**e,f**) Relative mRNA level of *Tnfα* and *Tgfβ* (E) and *Collagen 1α1* (F) measured by quantitative PCR in CTR and p38α^ΔH^ livers issued from mice treated or not by Ccl2/Ccl5 antibodies and its quantification at 40 hours post-CCl_4_. Gene expression levels were normalized to the abundance of *18 s* mRNA for each sample. Data represent the mean ± SEM (*n* ≥ 3 per group). *p < 0.05, **p < 0.01, ***p < 0.001 (two-tailed t-test).

To finally prove that the recruitment of the immune cells mediated the hepatoprotective response driven by p38α deletion, we blocked Ccl2/Ccl5 signals using specific neutralizing antibodies 5 hours before CCl_4_ exposure (Fig. 6a). We validated the effect of antibodies blockade by counting immune populations extracted from the livers and found a drastic decrease in the total number of immune cells (Fig. 6b) in both groups of mice. In the meantime, we showed that antibody blockade provoked a dramatic abolishment of hepatoprotection in p38α^ΔH^ livers through an amplification of necrotic regions (Fig. 6c) associated with a reduced anti-oxidative response (Fig. 6d). Moreover, we also found an accentuation of liver injury in control mice (Fig. 6c), suggesting that these hepatoprotective immune cells were already present in p38-proficient livers (Fig. 6b) but were massively recruited under p38α deficiency. Interestingly, we found a clear reduction in the level of *Tnfα* and *Tgfβ* transcripts (Fig. 6e) in both groups of mice concomitantly upregulated at 40 hours post-CCl_4_ challenge after Ccl2/Ccl5 blockade (Fig. 6e,f). These findings indicated that the combination of these two signaling (Tnfα and Tgfβ) participate to the hepatoprotective response. Accordingly, downregulation of *Collagen* 1α1 level was also observed after Ccl2/Ccl5 blockade (Fig. 6f), confirming the attenuation of liver tissue repair.

Altogether, our data clearly demonstrated the crucial requirement of these two chemotactic signals favoring the recruitment of immune cells to mediate the hepatoprotective response driven by p38α ablation.

## Discussion

Drug-induced liver injury and acute liver failure (ALF) remains a major problem in Western societies^45,46^. While significant progress has been made in the understanding of intracellular signaling mechanisms of toxicity related to various compounds in hepatocytes, e.g. paracetamol^47^, there is still an urgent need to develop potent therapeutic strategies to circumvent ALI and ALF. ALI can be studied in animal models and in isolated hepatocytes and most mechanisms are translatable to humans^27,48^. Due to its strong ability to integrate a variety of signaling pathways, previous reports highlighted p38 Mitogen Activated Protein Kinases (MAPKs) as potential appealing targets to improve ALI outcome. Most of these studies were done by using a hepatospecific ablation of p38α isoform arising either during foetal (hepatoblasts) or neonatal (immature hepatocytes) development. As p38α plays a crucial role in cellular differentiation^18,49,50^, we supposed that earlier deletion could impaired differentiation of hepatocytes and terminal liver maturation. In the present study, we developed a new inducible and hepatospecific mice model in which p38α isoform was completely deleted in mature hepatocytes. Until now, p38α ablation in the liver was shown as deleterious in different models of liver injury^22,25,51^. In this study, using the CCl_4_ model of acute liver injury, we demonstrate for the first time that p38α deletion generated a pro-hepatoprotective response against liver injury. Remarkably, we showed that p38α deficiency after CCl_4_ exposure, shaped the inflammatory response to promote efficient tissue repair. Finally, we evidenced that hepatoprotective response driven by p38α ablation was critically dependent on Ccl2/Ccl5 chemotactic signals, as their blockade dramatically exacerbated liver injury.

Following injuries, p38α MAPK displayed a wide range of cellular responses to ensure the maintenance of tissue homeostasis. Due to its major role as a negative regulator of cellular proliferation^20,21,36^, we expected to observe an extensive enhancement of hepatocytes proliferation secondary to p38α ablation. Differently, we found a lower proliferative response, reflecting buffered injuries (concomitant decrease in necrotic areas and ALT levels) in p38α^ΔH^ liver as compared to control one. Importantly, our data showed that p38α ablation did not increase the proliferation of hepatocytes after CCl_4_ exposure and also revealed that the function of p38α as a cell cycle checkpoint does not account for the hepatoprotective effect. Our findings are quite novel, since increased proliferation has been until now considered a hallmark of p38α deficient cells^23^.

Interestingly, we demonstrated that the deletion of p38α isoform in adult hepatocytes has strong repercussions on the immune microenvironment to mediate a potent hepatoprotective response favoring efficient hepatic tissue repair. Indeed, we found a drastic infiltration of immune cells mediated by Ccl2/Ccl5 chemokines. In addition, we clearly identified that Ccl2/Ccl5 chemotactic signals were crucial in that response as their neutralization sensitized to increase liver injury. Therefore, our findings highlight a new aspect in the pleiotropic role of p38α in hepatocytes during acute liver injury, as until now the beneficial effect of p38α deletion was strictly observed when performed in immune effectors such as liver myeloid cells or T/NKT cells^51,52^. Furthermore, the work of Kang and collaborators provided evidence that p38α ablation in hepatocytes was fueled by a drastic accentuation of liver injury associated with a massive inflammatory cell recruitment^51^. This study was conducted using a different model of acute liver injury (e.g. ConA). Collectively, these data underlie that the nature of stimuli-induced injury greatly influences the cellular response of p38α, as it does not trigger the same immune effectors involving preferentially either myeloid cells (e.g. CCl_4_, APAP) or lymphoid T cell reservoir (ConA)^53^. Therefore, it turns out that depending on the initial stimuli, the flavor of the inflammatory response dictates the outcome of tissue response. Our work clearly strengthens the critical connection between hepatocyte and immune system during acute liver injury and calls into question about the nature of the effectors involved in hepatoprotection. Further experiments are required to elucidate the molecular support of this dialogue. It is noteworthy that we found that the antioxidative response was determinant in the mediation of hepatoprotection in p38α^ΔH^ liver. Interestingly, Ccl2/Ccl5 antibody blockade considerably diminished this antioxidative response in our model. These observations are of importance as they illustrated that immune system could behave also as an additional partner to adapt the redox balance during liver injury^54^.

Finally, due to very limited therapeutic options for the treatment of acute liver injury, our work provides another field of treatment targeting specifically p38α in hepatocyte and manipulating immune response.

## Materials and Methods

### Generation of conditional knockout mice and animal care

Mice carrying two loxP sites flanking (floxed) exons 2 and 3 of the p38α gene (p38α^fl/fl^)^30^ were interbred with TTR-Cre-Tam mice expressing a tamoxifen-inducible Cre recombinase under the control of the hepatocyte-specific transthyretin promoter^31^ to generate p38α^ΔH^ mice (p38α^fl/fl^ TTR-Cre^+^-Tam) on the C57Bl6J genetic background. In all experiments, littermate carrying the respective loxP-flanked alleles but lacking expression of the Cre recombinase were used as controls (p38α^fl/fl^ TTR-Cre^−^-Tam). Mice were maintained at a constant temperature and humidity in light-controlled room with a 12 hours light cycle. They had free access to food (SAFE Laboratory) and tap water. To induce specific hepatocyte p38α deletion, four weeks old male p38α^ΔH^ were fed with tamoxifen diet (+1000 mg/kg TAM A115-T7100, Ssniff, Germany) during five days as well as their control littermates (p38α^fl/fl^ TTR-Cre^−^-Tam). All experiments were conducted in accordance with the institutional guidelines and the recommendations for the care and use of laboratory animals put forward by the Directive 2010/63/EU. This revises Directive 86/609/EEC on the protection of animals used for scientific purposes. All animal studies were approved by the Ministère de l’Enseignement Supérieur, de la Recherche et de l’Innovation (MESRI) and the Direction Départementale des Services Vétérinaires de Paris (agreement No. 75–956) and by the Mouse Facility Core laboratories (Institut Cochin, Inserm U1016/ CRC UMRS1138).

### Acute liver injury model

Experiments were performed both on control (p38α^fl/fl^ TTR-Cre^−^-Tam) and p38α^ΔH^ (p38α^fl/fl^ TTR-Cre^+^-Tam) male mice between 8 and 10 weeks of age. CCl_4_ (Merck, Germany) dissolved with sunflower oil [1:9] was administered intraperitoneally (IP) at 0.56 g/kg of body weight. Two hours before tissue harvest, mice were intraperitoneally injected with 50 mg/kg of Bromodeoxyuridine (BrdU) (Merck, Germany). Mice were euthanized at 0, 12, 24, 40, 48, 60 and 72 hours post-CCl_4_. After sacrifice, part of liver tissue was fixed in 4% neutral buffered formalin for immunohistochemistry analysis. The remaining liver tissue was flash frozen in liquid nitrogen and stored at −80 °C until used.

### Antibody depletion experiment

Five hours before CCl_4_ treatment, mice received a single IP injection of a cocktail of anti-Ccl2 (clone 2H5, Bio X Cell) antibody at a dose of 7.5 mg/kg and anti-Ccl5 antibody (clone 53405, R&D systems) at a dose of 1.0 mg/kg or control antibody (Polyclonal Armenian Hamster IgG, Bio X Cell; Normal Rat IgG control, R&D Systems). The efficacy of antibody depletion was evaluated 40 hours after CCl_4_ treatment.

### Serum Transaminase activity

Blood was collected from intracardiac puncture on anesthetized mice during time-course kinetic after CCl_4_ treatment and the activity of serum alanine aminotransferase [ALT] was measured using the AU400 chemistry analyzer (Olympus) (Biochemistry Facility, CRI Institute, Paris, France).

### Nonparenchymal cell isolation

As previously described^55^, livers were harvested and perfused with Hank’s balanced salt solution (1X HBSS) containing 10 mM HEPES, to remove circulating blood cells. The liver was passed through a stainless steel mesh in RPMI 1640 supplemented with 2% heat-inactivated fetal calf serum (FCS) (Gibco, ThermoFisher Scientific), 5 mM HEPES, 2 mM Glutamax (Gibco, ThermoFisher Scientific), 100 U/mL penicillin, 100 µg/mL streptomycin, and 5 × 10^−5^ M β-mercaptoethanol (Gibco, ThermoFisher Scientific). The liver cell suspension was collected and parenchymal cells were separated from nonparenchymal cells (NPCs) by centrifugation for 3 min at 800 rpm. The supernatant containing the NPCs was collected and centrifuged for 10 min at 1500 rpm. The pellet was then resuspended in 35% Percoll (GE Healthcare) diluted in RPMI 1640 supplemented with 2% FCS, 20 min at room temperature, at 2,000 rpm. The NPC fraction was collected at the bottom and the cells were collected by two rounds of centrifugation in ice-cold PBS. Red blood cells were removed by incubation with lysis buffer ACK (0.15 M NH4Cl, 10 mM KHCO3 and 0.1 mM Na2EDTA, pH 7.2). Cells were then washed in RPMI 1640 containing 10% FCS and centrifuged for 10 min at 1,500 rpm. Cells were resuspended in serum-containing medium and viable NPCs were counted by a trypan blue exclusion method, and stored on ice until further use.

### Gene expression analysis

Total RNA from mouse liver tissue was extracted using Trizol (ThermoFisher Scientific). Purified RNA was then reverse-transcribed with the High-Capacity cDNA Reverse Transcription Kit (Applied Biosystems). Quantitative PCR (q-PCR) was performed using a SYBR Luminaris Color HiGreen qPCR master mix (ThermoFisher Scientific) and specific primers (see Table 1). The reactions were performed in 96-well plates in a LightCycler 480 instrument (Roche) with 40 cycles. We determined the relative amounts of the mRNAs studied by means of the second-derivative maximum method, with LightCycler 480 analysis software and 18 s mRNA as the invariant control for all studies.

**Table 1:** Primer Sequences.

Gene	Forward	Reverse
18S	GTA-ACC-CGT-TGA-ACC-CCA-TT	CCA-TCC-AAT-CGG-TAG-TAG-CG
Catalase	ACA-TGG-TCT-GGG-ACT-TCT-GG	CAA-GTT-TTT-GAT-GCC-CTG-GT
Ccl2	TCT-GGG-CCT-GCT-GTT-CAC-A	GGA-TCA-TCT-TGC-TGG-TGA-ATG-A
Ccl5	GCT-GCT-TTG-CCT-ACC-TCT-CC	TCG-AGT-GAC-AAA-CAC-GAC-TGC
Collagen 1α1	GAG-CGG-AGA-GTA-CTG-GAT-CG	GCT-TCT-TTT-CCT-TGG-GGT-TC
Collagen 3α1	GAA-GTC-TCT-GAA-GCT-GAT-GGG	TTG-CCT-TGC-GTG-TTT-GAT-ATT-C
Cyp2e1	CGT-TGC-CTT-GCT-TGT-CTG-GA	AAG-AAA-GGA-ATT-GGG-AAA-GGT-CC
Gstm3	TAT-GAC-ACT-GGG-CTA-TTG-GAA-CAC	GGG-CAT-CCC-CCA-TGA-CA
Ho1	AAG-CCC-AGA-ATG-CTG-AGT-TC	GCC-GTG-TAA-TAT-GGT-ACA-AGG-A
IL1β	GCC-CAT-CCT-CTG-TGA-CTC-AT	AGG-CCA-CAG-GTA-TTT-TGT-CG
Nrf2	AGG-ACA-TGG-AGC-AAG-TTT-GG	TCT-GTC-AGT-GTG-GCT-TCT-GG
Tgfβ1	TGG-CGT-TAC-CTT-GGT-AAC-C	GGT-GCT-GGG-CCC-TTT-CCA-G
Tnfα	CAT-CTT-CTC-AAA-ATT-CGA-GTG-ACA-A	TGG-GAG-TAG-ACA-AGG-TAC-ACC-CC

### Western blotting analysis

Total proteins were extracted from snap-frozen livers as described previously^56^. Protein concentration was determined using the bicinchoninic acid assay (Bio-Rad Protein Assay). Proteins (40 μg) were resolved by SDS-PAGE and then transferred onto nitrocellulose membranes (0.45-μm pore size), which were incubated overnight at 4 °C with primary antibodies. Primary antibodies used for western blotting are referenced in Table 2. The proteinbound primary antibodies were detected with an appropriate horseradish peroxidase–conjugated secondary antibody (ThermoFisher Scientific). Immunoreactive bands were revealed using the “Clarity Western ECL Substrate” purchased from Bio-Rad. Blots were exposed to Amersham Hyperfilm (GE Healthcare Life Sciences). In all immunoblotting, HSC70 was used to normalize the results. For protein quantification, densitometry analysis was performed using Image J 1.8.0_112. Data are presented as relative units, which represent the densitometric value for the phosphoprotein of interest that was normalized to the total levels of the same protein.

**Table 2:** Antibodies used in immunohistochemistry or western blot experiments.

Antibody	Dilution	Manufacter and Reference
BrdU	1/400	Thermo Fisher #MA5-12502
Cleaved-Caspase 3	1/100	Cell Signaling #9664
Cyclin A2	1/2000	Abcam #32386
Cyclin B1	1/1000	Cell Signalling #4138
Cyclin D1	1/3000	Pierce MA1-39546
HSC70	1/25000	Santa Cruz #7298
p38α	1/4000	ThermoFisher Scientific #PA5-17713
PHH3	1/500	Millipore #06-570
phospho-p38	1/4000	Cell Signalling #4511

### Histology, immunohistochemistry

Tissue was fixed by incubation in 4% formol overnight at 4 °C and embedded in paraffin wax. Hematoxylin/eosin staining was carried out on 5-µm paraffin sections. For immunohistochemistry, liver sections (5 µm) were de-paraffinized and incubated in citrate buffer at 95 °C for 20 min for antigen retrieval. Sections were treated with 3% hydrogen peroxide for 15 min at room temperature and then incubated overnight at 4 °C with the primary antibodies referenced in Table 2. After three washes in PBS1X, tissue sections were incubated with biotinylated anti-mouse/rabbit or rat IgG (1/200 dilution, Vector Laboratories, CA, USA) for 1 hr at RT and then washed three times in PBS1X, after which streptavidin–horseradish peroxidase conjugates (Vector Laboratories, CA, USA) were added and the slides incubated for 45 min. After three washes with PBS1X, DAB solution (Vector Laboratories, CA, USA) was added and the slides were counterstained with haematoxylin.

### *In situ* detection of ROS

Fresh cross sections (8 μm) of unfixed, frozen mouse livers were immediately incubated with 5 μM DHE at 37 °C for 30 minutes in a humidified chamber, subsequently washed twice with ice-cold phosphate-buffered saline, and coverslipped^57^. The fluorescence intensity of DHE staining was measured with ImageJ software.

### Image acquisition and analysis

Concerning HE, BrdU and PHH3 labelling, images were taken using a Nikon Statif Eclipse E600 microscope with x10 and x20 magnification, 1.4–0.7 NA PL-APO objectives, a DXM1200 cooled CCD camera (Nikon), and ACT-1 (version 2.63; Universal Imaging). For cleaved-caspase 3 labelling, images were taken using an Olympus BX63F, at 4x magnification Uplan FLN objective, an Olympus DP73 camera and Metamorph software. Necrotic area were quantified by morphometric analysis using an open-source ImageJ software in 5 fields at x10 magnification. For BrdU/PHH3 staining, 4000 hepatocytes (for each liver sample analyzed) were counted; at least 10 areas of 33,500 μm^2^ were analyzed. Cleaved-caspase 3 immunostaining was quantified by color segmentation using an open-source ImageJ software in 5 fields at 4x magnification. Adobe Photoshop CS (Adobe Systems Software) was used for figure construction.

### Statistical analysis

Statistical significance was determined with a 2-tailed Student’s t test performed using GraphPad Prism 6.0 (GraphPad Software Inc). All data are representative of 3 to 10 animals of each genotype and are expressed as mean ± SEM. A P value of less than 0.05 was considered statistically significant.

## Supplementary information

SUPPLEMENTARY FIGURES
