# Supplementary material for: Hepatospecific ablation of p38α MAPK governs liver regeneration through modulation of inflammatory response to CCl4-induced acute injury
Source: Sci Rep. 2019 Oct 10;9:14614. doi: 10.1038/s41598-019-51175-z (PMC6787013; doi:10.1038/s41598-019-51175-z)
Supplement: Supplementary file 1 — SUPPLEMENTARY FIGURES [file 41598_2019_51175_MOESM1_ESM.pdf]

Hepatospecific ablation of p38α MAPK governs liver regeneration through modulation of inflammatory response to CCl_4-induced acute injury

Manon Fortier[1,2], Mathilde Cadoux[1,2], Nadia Boussetta[2], Sandrine Pham[1,2], Romain Donné[1,2], Jean-Pierre Couty[1,2], Chantal Desdouets[1,2, §] and Séverine Celton-Morizur[1,2, §, *].

1 Centre de Recherche des Cordeliers, INSERM, Sorbonne Université, USPC, Université Paris Descartes, Université Paris Diderot, Team « Proliferation, Stress and Liver Physiopathology », F-75006 Paris, France.

2 INSERM, U1016, Institut Cochin, Paris, France ; CNRS, UMR8104, Paris, France.

* Corresponding author : Dr. Séverine Celton-Morizur, Centre de Recherche des Cordeliers, INSERM UMRS 1138. Email: severine.morizur@inserm.fr.

§Authorship note: Chantal Desdouets and Séverine Celton-Morizur contributed equally to this work.

Supplementary figures

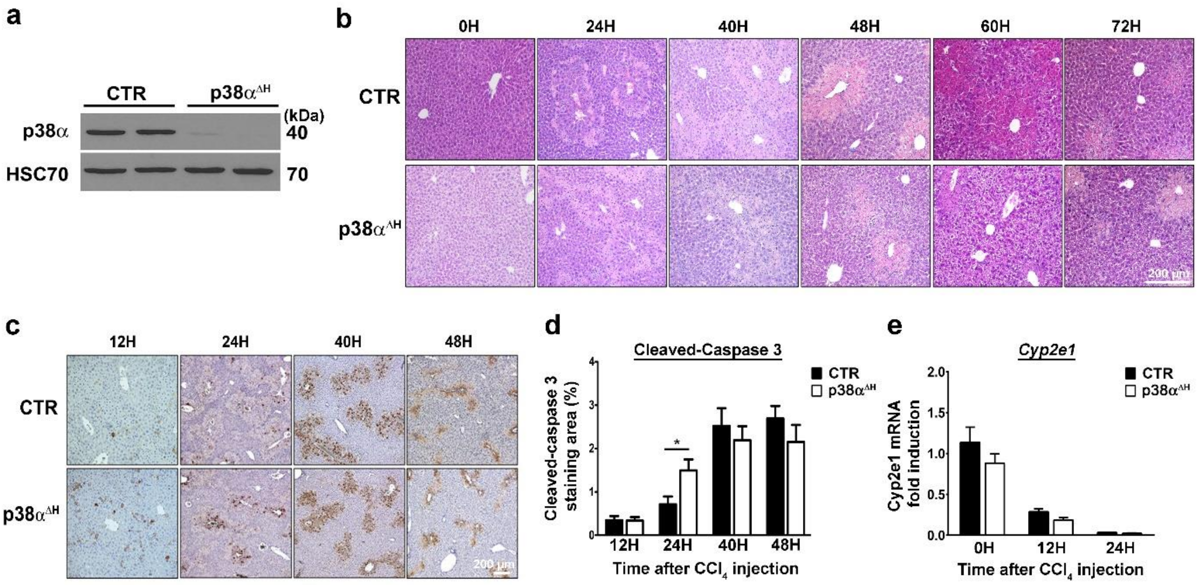

Supplementary Figure 1. Apoptosis and CCl_4 bioactivation are not impaired in p38α$^{\Delta H}$ mice during acute liver injury.

a) Immunoblot analysis of p38α isoform in CTR and p38α$^{\Delta H}$ liver samples in steady state conditions. HSC70 served as a loading control. Lanes show samples from independent biological replicates. The displayed figure was cropped and the original images are part of the supplementary data.

b) Magnification of necrotic area related to Figure 2b, in CTR and p38α$^{\Delta H}$ livers at different times after CCl_4 exposure.

c) Representative Cleaved-Caspase 3 immunochemistry of CTR and p38α$^{\Delta H}$ liver sections at indicated time points after CCl_4 injection.

d) Quantitative analysis of Cleaved-Caspase 3 labeled CTR and p38α$^{\Delta H}$ liver sections (percentage of brown area). Data represent the mean ± SEM ($n \geq 4$ per group). *$p<0.05$(two-tailed t-test).

e) Relative mRNA level of *Cyp2e1* measured by quantitative PCR in CTR and p38α$^{\Delta H}$ liver samples before (0H) and after CCl_4 injection (12H-24H). Gene expression levels were normalized to the abundance of *18s* mRNA for each sample. Data represent the mean ± SEM ($n \geq 6$ per group).

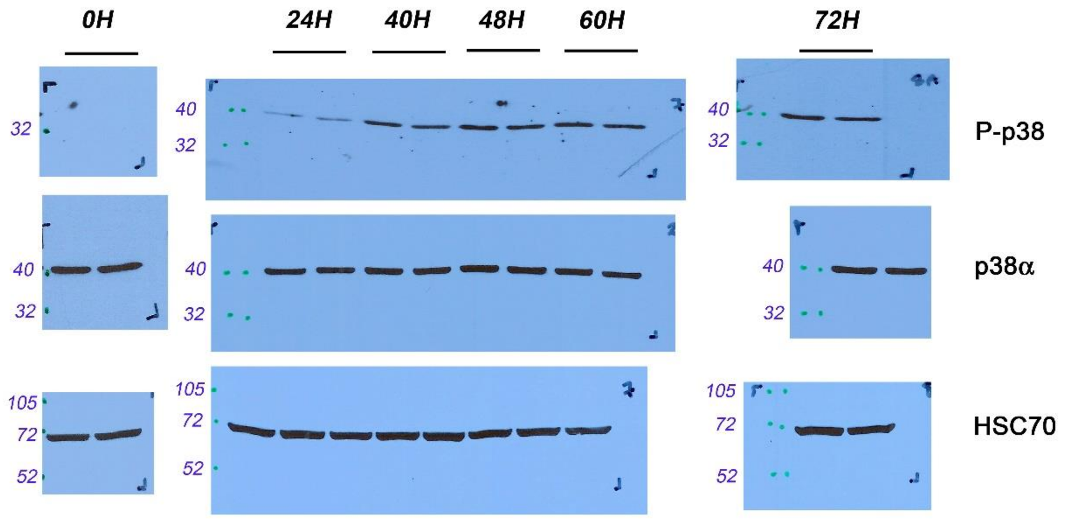

Supplementary Figure 2. Original pictures from Western blot membranes related to Figure 1b.

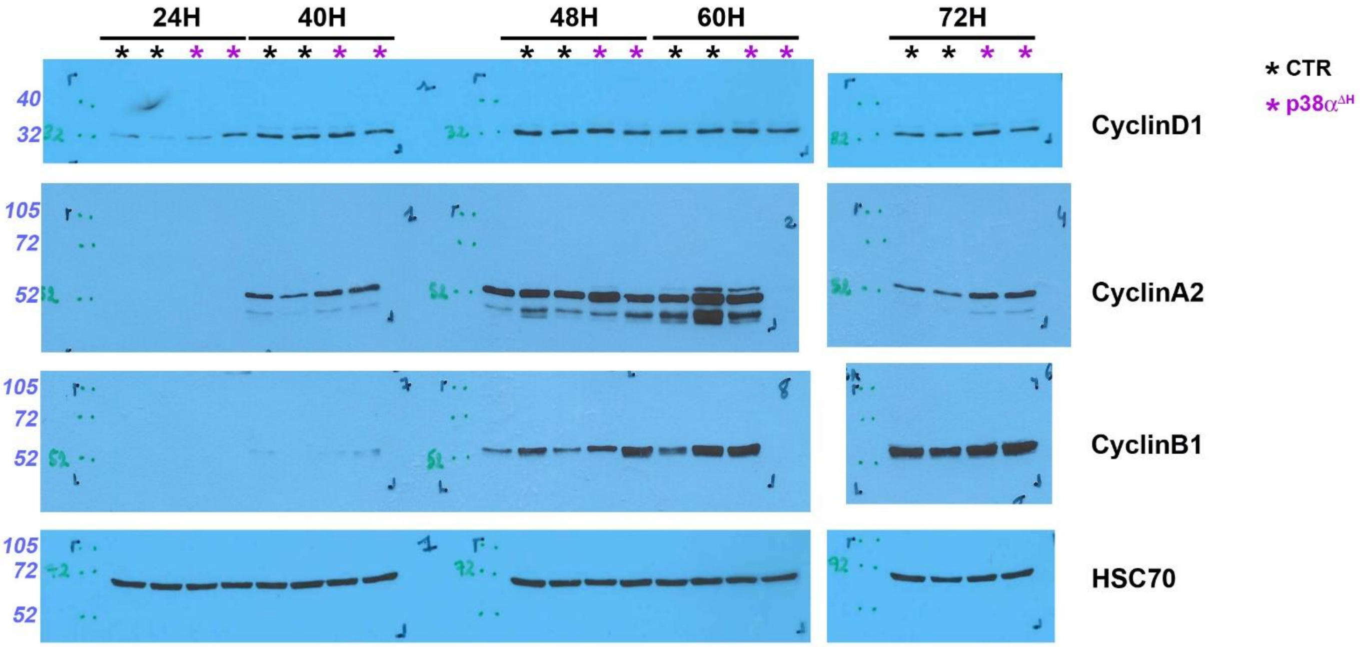

Supplementary Figure 3. Original pictures from Western blot membranes related to Figure 3e.

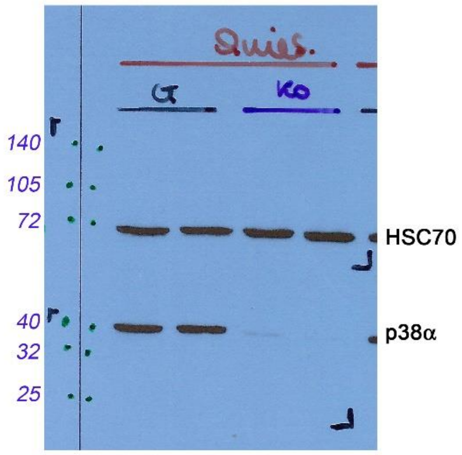

Supplementary Figure 4. Original pictures from Western blot membranes related to Figure Supplementary 1a.
